# Repopulation of tumor cells during fractionated radiotherapy and detection methods (Review)

**DOI:** 10.3892/ol.2014.1990

**Published:** 2014-03-21

**Authors:** JIA YANG, JIN-BO YUE, JING LIU, JIN-MING YU

**Affiliations:** 1 Shandong Cancer Hospital and Institute, Jinan University, Shandong Academy of Medical Sciences, Jinan, Shandong 250117, P.R. China; 2 Department of Radiation Oncology, Shandong Cancer Hospital and Institute, Jinan, Shandong 250117, P.R. China; 3 Graduate Education Centre of Shandong Academy of Medical Sciences, Jinan, Shandong 250117, P.R. China

**Keywords:** tumor repopulation, proliferation, fractionated radiotherapy, functional imaging, cancer stem cell

## Abstract

Repopulation of tumor cells during radiotherapy is believed to be a significant cause for treatment failure. The phenomenon of tumor repopulation during fractionated radiotherapy was found from clinical observations that identified that the local control rate decreased with a prolonged treatment time. A series of animal experiments with varied overall treatment time and fractionated doses were performed to demonstrate tumor cell repopulation during radiotherapy in various mouse xenograft models. However, conventional detection methods are challenging, as it is difficult to separate viable cells from those destined for apoptosis during fractionated radiotherapy. In essence, the mechanism of tumor repopulation involves the continuing proliferation of clonogenic tumor cells. *In vivo* imaging, tracking and targeting of the repopulation of these cells has been of clinical interest so as to administer a higher dose to the tumor repopulation regions. Currently, functional imaging methods, including 3′-deoxy-3′-^18^F-fluorothymidine positron emission tomography (^18^F-FLT PET), are showing promise in assessing the proliferation activity of tumors *in vivo*. This review mainly focuses on the phenomenon of tumor repopulation during radiotherapy and its conventional and novel detection methods, particularly on the feasibility of ^18^F-FLT PET for the detection of tumor-cell repopulation.

## 1. Introduction

The process by which surviving tumor cells have the ability to continue proliferation during fractionated radiotherapy is known as tumor repopulation, as the cells are able to regenerate the tumor. There is evidence that the repopulation of tumor cells can lead to radioresistance and limit the effectiveness of radiotherapy (1,2). Conventional methods for detecting tumor-cell repopulation include tumor control probability, 50% tumor control dose (TCD50), potential doubling time (Tpot) and pathological proliferation parameters. However, it is difficult to separate viable cells from those destined for apoptosis during fractionated radiotherapy using these methods. *In vivo* imaging, tracking and targeting the repopulation of clonogenic tumor cells has been of clinical interest, with the aim to administer a higher dose of radiotherapy to repopulation regions through use of intensity-modulated radiotherapy (IMRT). Functional imaging tracers, such as 3′-deoxy-3′-^18^F-fluorothymidine (^18^F-FLT), can reflect the proliferation of tumor cells by showing the activity of thymidine kinase, and have been a useful tool in estimating proliferation and predicting the response of increased sensitively during radiotherapy (3,4). In the present review, the demonstration of tumor-cell repopulation, the molecular imaging of tumor stem cells and the non-invasive, quantitative functional imaging for detecting tumor repopulation during fractionated radiotherapy is explored.

## 2. Demonstration of tumor repopulation during radiotherapy

Tumor repopulation, a hypothetical mechanism, was found in clinical practice and was then demonstrated by animal experiments and further corroborated by clinical studies. Conventional fractionated radiotherapy is delivered in small doses (1.8–2.0 Gy), which are often administered daily on weekdays with 5 fractions a week. The reason for this schedule is to allow the recovery of normal tissues from sublethal radiation damage between treatments and to avoid severe toxic reactions; however, repopulation of surviving tumor cells may also occur (2). A series of clinical studies identified that in certain types of cancer, including tonsilar fossa, bladder and cervical cancer, the tumor control rate decreased dramatically when overall treatment time was prolonged (5–7). A study by Withers *et al* (8) found that rapid tumor regrowth occurred during extensions of radiotherapy treatment from ~5–8 weeks in almost 500 patients with oropharyngeal cancer. The study concluded that clonogen repopulation in squamous cell carcinomas of the head and neck accelerates following a lag period of 4±1 weeks subsequent to the initiation of radiotherapy. The existence of the significant time factor for tumor tissues during fractionated radiotherapy has also been corroborated by a large number of randomized phase III trials, which demonstrated an impact of overall treatment time on local control for head and neck, non-small cell lung and esophageal cancers (9–11). There are also certain studies that have used the linear-quadratic model to estimate tumor repopulation rate and its onset time during radiotherapy, and to measure the extra radiation dose required to compensate for the additional duration of the treatment (12–14).

The existence of the significant time factor stimulated basic research in the laboratory, with the aim to demonstrate tumor repopulation during radiotherapy. A series of animal experiments with varied overall treatment times were performed in various mouse xenograft models, which included human FaDu squamous cell carcinoma (15), human melanoma (16), human soft tissue sarcoma (17) and MCA-4 mammary carcinoma (18). In these experiments, the parameters, including TCD50, Tpot or S phase fraction (SPF), were used to reflect the proliferation activity. Similar results showed that the tumor control rate decreased as the overall treatment time increased. The extra radiation dose was required to compensate for the additional duration of treatment. Thus, tumor repopulation was inferred from the extra radiation dose and decreased the tumor control rate.

It is now generally accepted in clinical practice that prolongation of overall treatment time can lead to tumor repopulation and it is essential that is it avoided. At present, accelerated fractionation schedules have been widely accepted by clinicians to shorten the overall treatment time of radiotherapy in order to counteract tumor-cell repopulation (9,11). Additionally, a study by Gao *et al* (1) recently proposed a cellular Potts model that simulates the kinetics of glioma stem cells (GSCs) and non-stem cancer cells (CCs) in glioblastoma growth and radiation response. The study found that CCs die and GSCs become enriched and potentially increase in number during each fraction of radiation, which may lead to accelerated repopulation following fractionated radiation treatment.

A study by Petersen *et al* (19) proved tumor repopulation using a typical animal experiment during fractionated radiotherapy with pathological validation. In the study, human FaDu squamous cell carcinomas in nude mice were irradiated daily or every second day with 12–18 fractions, 3 Gy per fraction. At various time points, the tumors were excised and then stained for Ki-67 and bromodeoxyuridine (BrdUrd), and the labeling indices were shown to initially decrease and then increased again at later times during the course of the fractionated radiotherapy. The staining intensity of the epidermal growth factor receptor (EGFR) produced a similar kinetic pattern, and the histological results were notably matched with the kinetics of clonogenic tumor cell repopulation. Several other animal models, including mouse fibrosarcoma and mouse ovarian tumor (20,21), and clinical studies based on human breast carcinoma and rectal cancer (22,23), also revealed similar results that Ki-67, BrdUrd labeling indices or SPF decreased initially and increased again at a later time during the course of radiotherapy. Again, the histological results were consistent with the kinetics of clonogen repopulation.

## 3. Conventional methods for detecting tumor repopulation during radiotherapy

Conventional methods of measuring tumor repopulation are based on tumor volume or diameter changes measured visually, Ki-67 and BrdUrd detection by immunohistochemical staining (19) or SPF and Tpot determined by flow cytometry (24). These methods have been shown to provide useful clinical information in various human cancers and, notably, the pathological diagnosis is the gold standard that indicates the presence or absence of cancer, the type of cancer and its classification. It is desirable that tumor repopulation could be proven by proliferation parameters with Ki-67, BrdUrd or SPF. Methods of measuring tumor growth are less sensitive as tumor repopulation may happen independent of tumor diameter or volume change underlining the mechanisms of cell loss decrease, a difference in cell repair and cell reoxygenation. The anticancer effect of radiotherapy is applied through the accumulation of DNA damage in the tumor cells, which may result in acute or delayed cell death known as mitotic catastrophe. Therefore, measuring tumor cell proliferation, as assessed by the uptake of markers of DNA synthesis, such as BrdUrd, or by using flow cytometry to measure DNA content, may not distinguish viable cells from those destined to die during fractionated radiotherapy (25). Also, immunohistochemical staining methods require tissue samples and are therefore invasive and limited by sampling variability. In a recent study by Gerlinger *et al* (26), it was identified that intratumor heterogeneity using immunohistochemical analysis, mutation functional analysis and profiling of mRNA expression may lead to underestimation of the genomics as depicted from a single tumor-biopsy sample and may create major challenges for personalized medicine and biomarker development.

Therefore, identifying a functional imaging technology that is non-invasive, accurate, well reproducible and in particular can detect the proliferation activity and therapeutic efficacy *in vivo* has become a hot research topic.

## 4. Detecting proliferation of clonogenic CSCs with non-invasive molecular imaging technology

In essence, the mechanism of tumor repopulation involves the continuing proliferation of clonogenic cancer stem cells (CSCs). In 2012, three studies were published that used a genetic cell-labeling technique to monitor the proliferation of CSCs (27–29). It has been shown that CSCs in the brain, skin or intestinal tumors are indeed the source of tumor regrowth (27). *In vivo* imaging, tracking and targeting of the proliferation activity of CSCs appears to be of significance.

Molecular imaging is a novel and non-invasive strategy that allows real-time monitoring of CSCs, which are believed to be responsible for tumor development, metastasis and relapse following conventional therapy (30) *in vivo*, through use of various molecular-targeted imaging probes that are specific for cell surface biomarkers. Evidence that various solid tumors are organized by hierarchy and maintained by a clear subpopulation of CSCs is increasing. Pioneering studies using spontaneous mouse leukemias and lymphomas have identified that the frequency of tumor-propagating cells can range from 1% to the majority of cells (31,32). A study by Leyton *et al* (33) revealed a humanized radioiodinated minibody as a positron emission tomography (PET) imaging agent for the detection of prostate stem cell antigen-positive prostate cancer. A study by Yoshii *et al* (34) proposed Cu-64-diacetyl-bis (N4-methylthiosemicar bazone) (Cu-64-ATSM) as a PET imaging agent for the detection of cluster of differentiation 133^+^ (CD133^+^) CSCs. Tsurumi *et al* (35) showed that a CD133-specific monoclonal antibody, AC133.1, may be used for quantitative fluorescence-based optical imaging of mouse xenograft models.

A recent study from Vlashi *et al* (36) revealed that 72 h following irradiation with 5×3 Gy in a human glioma model, there was an increase in the percentage of CSCs. The study used the absence of 26S proteasome activity as a marker for monitoring CSCs and implemented modern real-time imaging techniques. The percentage of proliferating cells was also measured by an increase in Ki-67 to a higher extent in marker-positive vs. marker-negative cells, which are interpreted as an effect of repopulation of CSCs. The development of molecular imaging for tracking CSCs *in vivo* may provide the possibility of detecting repopulation of clonogenic CSCs. However, concerns remain for the limitations of a clinical imaging technique, such as PET, with a limited spatial resolution for the detection of clonogenic CSCs, even if an appropriate molecular imaging tracer exists, as in certain cases CSCs may constitute <1% of the tumor population (37).

There remain numerous unresolved problems, despite the substantial evidence for the existence of CSCs in mouse and human carcinomas. High-resolution imaging technology together with stromal markers shows promise and will improve the understanding of the cellular niche for various CSCs.

## 5. PET tracers for imaging tumor-cell proliferation *in vivo*: Current status

PET is a molecular imaging technique that can provide various quantitative measurements of the underlying tumor biology, depending on the radiotracer used. The radiotracer is injected through a vein, accumulates in the tumor and the radioactive emissions are detected by the PET camera. PET tracers, including 2-^11^C-thymidine, ^76^Br-bromofluorodeoxyuridine (^76^Br-BFU) and ^11^C-2′fluoro-5-methyl-1-β-D-arabinofuranosyluracil (^11^C-FMAU), are labeled nucleotides that are directly incorporated into DNA. The thymidine analog, 3′-deoxy-3′-fluorothymidine (FLT), is currently the most widely used radiotracer, and all are considered to be proliferation markers and are useful additions to the imaging, which can provide additional diagnostic specificity and biological information for treatment planning and response monitoring. However, the short half-life of ^11^C (20.4 min) and the rapid catabolism of thymidine following injection results in 2-^11^C-thymidine being less conducive for routine clinical use. Compared with ^11^C, ^18^F has a longer half-life (109.8 vs. 20.4 min) and is now generally accepted in clinical practice (38). Limitations of ^76^Br-BFU are the necessity for co-injection with cimetidine, the rather cumbersome production of ^76^Br and the high radiation dose of ^76^Br (half-life of 16 h) as compared with ^11^C or ^18^F (38). The main limitation of ^11^C- or ^18^F-FMAU appears to be that it is a relatively poor substrate for thymidine kinase 1 (TK1) and a relatively good substrate for TK2. The use of ^11^C- or ^18^F-FMAU is dependent on the extent to which tumor uptake is associated with TK1 vs. TK2 activity. ^11^C- or ^18^F-FMAU retention may be less sensitive in comparison with ^18^F-FLT retention in cell proliferation change (39). Therefore, the PET radiotracer, ^18^F-FLT, may show promise in assessing the proliferation activity of tumors *in vivo* and the feasibility in detecting tumor-cell repopulation.

## 6. Imaging proliferation of tumor cells via ^18^F-FLT labeling

FLT is a pyrimidine analog which, following uptake into the cell, is phosphorylated by TK-1 into ^18^F-FLT monophosphate, but is not directly incorporated into DNA, thus causing intracellular sequestration of radioactivity (3,4). TK-1 is the main enzyme in the salvage pathway of DNA synthesis, and increases in activity during the S phase of the cell cycle. ^18^F-FLT uptake, therefore, reflects the cell proliferation status (40). A study has shown that ^18^F-FLT, as a positron tracer reflecting cell proliferation, can be used in PET imaging to observe *in vivo* tumor cell proliferation at a molecular level, non-invasively, and quantitatively across the entire tumor. For pre-clinical studies, FLT uptake as a measurement of TK-1 activity correlates strongly with pathology-based cell proliferation measurements (41). For clinical studies, however, results are conflicting with certain studies demonstrating a good association between FLT and Ki-67 (42–44), whilst others present a negative association (45–47). Biological explanations for the absence of FLT/Ki-67 correlation include a loss of cell cycle-specific regulation of TK1 (48), cell adenosine triphosphate levels (48), FLT representing only the salvage pathway of thymidine metabolism (49) and difference in the phosphorylation rate between FLT and thymidine (45). In addition, the accuracy of the measurement of the biopsy samples will be subject to sampling errors and reduced reproducibility, as it does not take into account the degree of intratumor heterogeneity expression for this marker. Chalkidou *et al* (50) recently conducted a systematic review and meta-analysis of the correlation between FLT and Ki-67. The study attributed variations between FLT and Ki-67 to the methods used and the study design. Larger clinical studies with an improved study design are justified for validation of these findings for specific cancer types which have conflicting results.

^18^F-FLT PET has been reported to have more of a cancer-specificity for diagnosing malignancy compared with ^18^F-FDG PET in head and neck, pancreatic and esophageal cancer (51–53). There are several available studies in which ^18^F-FLT and ^18^F-FDG uptake have been compared in inflammatory tissues. The studies confirm that ^18^F-FLT is a more cancer-specific tracer and they indicate that fewer false-positive ^18^F-FLT PET scans occur in the patient (54,55). ^18^F-FLT PET has also been shown to be a more sensitive tool that can provide an early identification of tumor response for radiotherapy, chemotherapy or EGFR inhibitor drugs (56–58). It can also sensitively reflect proliferation of normal tissues during radiotherapy (56).

Notably, ^18^F-FLT PET has been reported to detect tumor repopulation during fractionated radiotherapy. A pilot clinical study using serial ^18^F-FLT PET/computed tomography (CT) scans to measure tumor proliferation has been performed by Yue *et al* (59). In the study, two patients out of 21 had unplanned interruptions of the radiotherapy treatment and then underwent ^18^F-FLT PET/CT scans, which had a corresponding increase in ^18^F-FLT uptake, indicating tumor repopulation. The classic understanding of repopulation is that it usually occurs following ~4 weeks of radiotherapy (8); however Fowler (60) indicates that repopulation begins earlier. Experimental data from a study by Schmidt-Ullrich *et al* (61) supports this hypothesis, showing that the molecular process of accelerated repopulation is mediated through radiation-induced EGFR activation, and it may occur following a single 2-Gy fraction. A study by Everitt *et al* (62) also observed a ‘flare’ of ^18^F-FLT uptake in primary non-small cell lung cancer following only 2 Gy irradiation.

In order for the use of ^18^F-FLT PET for detecting tumor repopulation to be accepted and introduced into clinical studies, validation with tumor histology is mandatory. A study by Fatema *et al* (63) evaluated the sequential changes in intratumoral proliferative activity in head and neck cancer xenografts (FaDu) using FLT. The study found that 6 h following radiation treatment, the intratumoral ^3^H-FLT level diffusely decreased and then subsequently increased gradually with time. This is consistent with the experimental results of tumor repopulation that was pathologically proven by Petersen *et al* (19). Other than this, there is no literature reporting the detection of tumor repopulation using functional imaging together with pathological validation. Also, CSCs markers, including CD44^+^ and CD133^+^, together with high-resolution imaging, will improve the understanding of tumor repopulation during fractionated radiotherapy.

**Figure 1:**
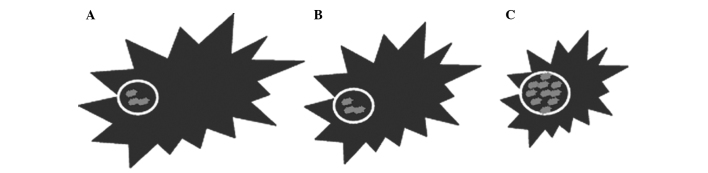
Models for the region of clonogenic tumor cell changes with tumor repopulation. (A) Region of clonogenic tumor cells prior to radiotherapy. (B) During radiotherapy, the volume of clonogenic tumor cells may shrink or remain unchanged due to its radioresistance. (C) Tumor continues to shrink, however, the residual tumor is reoxygenated due to improved oxygen and then repopulation occurs.

In addition, the reappearance of ^18^F-FLT uptake is of great interest for investigation of the association with the initial uptake prior to radiotherapy (Fig. 1). There is a difference in the spatial correlation between the region of clonogenic tumor cells in A and C in Fig. 1. If ^18^F-FLT PET/CT prior to treatment could predict potential tumor repopulation, then it could also be used to determine a biological target volume for radiotherapy. Thus, ‘dose painting’ via IMRT may be applied to escalate dose to repopulation regions.

## 7. Conclusion

The demonstration of tumor repopulation has developed from clinical observation to animal experiments and human cancer verification, which can be further corroborated and applied in a clinical practice. Functional imaging, as a non-invasive, quantitative method can be safely performed for any lesion and be repeated multiple times, permitting the evaluation of an entire tumor and providing information associated with the regional heterogeneity in a tumor during radiotherapy. Functional imaging, such as ^18^F-FLT PET, as a non-invasive, reliable and promising functional imaging technique has been a useful tool in oncology for estimating tumor proliferation change during radiotherapy, with more specificity and sensitivity. ^18^F-FLT PET may present as one of the potential molecular imaging modalities *in vivo* and for targeting the repopulation of clonogenic tumor cells during fractionated radiotherapy. Ongoing research based on pathology and modern real-time imaging techniques together with CSC markers for tracking of CSCs is underway in our institute to examine whether ^18^F-FLT PET can detect tumor repopulation during radiotherapy in nude mice and humans.
